# Parasitic threat in commercial organic fertilizers

**DOI:** 10.1007/s00436-022-07451-5

**Published:** 2022-02-04

**Authors:** Aleksandra Figura, Tomasz Cencek, Elżbieta Żbikowska

**Affiliations:** 1 Department of Invertebrate Zoology and Parasitology, Nicolaus Copernicus University, Toruń, Poland; 2 Department of Parasitology, National Veterinary Research Institute, Puławy, Poland

**Keywords:** Organic fertilizers, Sewage sludge, Nematode eggs

## Abstract

The use of fertilizers based on sewage sludge is common practice. Due to the possible presence of pathogens and eggs of intestinal parasites like *Ascaris* sp., *Toxocara* sp., and *Trichuris* sp. in these products, it is necessary to control them. The aim of the study was to determine the presence of parasite eggs in commercial organic fertilizers available on the market. Selected commercial products were tested using the Quinn flotation method and a method dedicated to the study of dewatered sewage sludge. Assessment of the viability of helminth eggs was carried out on the basis of staining with calcein and propidium iodide. In 57% of the tested samples, the presence of live eggs of the abovementioned parasites was detected, and in 21% of samples, the eggs with live larvae were detected. Eggs of *Trichuris* sp. (50%) and *Ascaris* sp. (36%) were the most common. The obtained results clearly indicate that the process of hygienization of the sewage sludge before the production of fertilizers was not effective enough and it is necessary to standardize the prophylaxis against the spread of parasitic nematodes in commercially available products.

## Introduction

In many countries, including Poland, there are legal regulations concerning the use of fertilizers and fertilization process. These acts contain the conditions and procedure for the commercialization of fertilizers. The manufacturer or importer submits full documentation on the quality of the product, test results of relevant samples, and the necessary opinions on the safety of the product. For example, in the USA, this problem is regulated by Federal Water Pollution Control Acts. In Poland, the authority issuing the permit for an indefinite period is the Ministry of Agriculture and Rural Development. This permit may be withdrawn if the quality requirements are not met or it is proven that the product poses risk to human or animal health. The basis for withdrawal is failure to meet the conditions set out in the regulation of the European Parliament (2019/1010).

The source of material to commercial purposes is, among others, sewage sludge. Total sludge production in China in 2013 was 6.25 million tons of dry solids and showed an annual growth of 13% from 2007 to 2013 (Yang et al. 2015). In the USA, 13.8 million tons of dry sludge per year was captured during wastewater treatment (Seiple et al. 2017). The amount of sewage sludge in the EU27 expected for 2020 exceeded 13 million tons (dry solids) (Milieu Ltd report, 2010). The global problem of the production and use of excreta, wastewater sludge, and biosolids is presented in detail by LeBlanc et al. (2008). In Poland, in 2012, sewage treatment plants produced 562 thousand tons of dry matter (DM) of sewage sludge (Bianchini et al. 2016). For several years, they have been used more and more often in various branches of the economy. According to Styka and Beńko (2014) in 2009–2011, 9,606,000 tons DM of sewage sludge was produced in Europe. Bianchini et al. (2016) indicated that of all sewage sludge produced in Europe in 2011, 47.4% was used in agriculture, 23.6% was burned, 9.5% was stored, 8.7% was processed for the production of fertilizers, and 10.7% for other purposes—e.g., forestry, melioration. The use of sewage sludge in agriculture and for the production of fertilizers is fraught with problems related to pathogenic organisms present in the sediments (Wolna-Maruwka et al. 2009). Pathogenic bacteria or mold fungi are detected on the basis of standard tests taking into account the cultivation methods on artificial substrates (Kosicka-Dziechciarek et al. 2015). The high efficiency of these tests guarantees the reliability of the obtained results. A more serious problem is the presence of eggs of the parasitic nematodes. The exceptional resistance to environmental factors of eggs of nematodes: *Ascaris* sp., *Trichuris* sp., *Toxocara* sp. (Hanjra et al. 2012), has determined the choice of having them as indicators to assess the hygienic condition of sewage sludge (Dąbrowska et al. 2014). The search for nematode eggs in the hygienized sewage sludge is described in detail in the relevant regulations (Rorat et al. 2019). However, the credibility of the obtained results largely depends on the quite subjective visual assessment (Włodarczyk et al. 2014). Based on the negative test for *Ascaris* sp., *Trichuris* sp., and *Toxocara* sp. (ATT) eggs; pathogenic bacteria; and heavy metals in the samples, all the sewage sludge can be used for the production of fertilizers. We set up a working hypothesis that the current procedures for testing the presence of parasitic eggs do not guarantee the safety of commercial organic fertilizers based on sewage sludge. In order to verify the hypothesis, we tested the presence of *Ascaris* sp., *Toxocara* sp., and *Trichuris* sp. eggs, and assessed their viability in commercial products for fertilizing plants.

## Material and methods

The 14 organic fertilizers were tested: (i) three organic fertilizers (made of an organic substance or mixtures of organic substances, including biohumus, compost by-crops), (ii) two organic-mineral fertilizers (mixture of organic and mineral fertilizers), (iii) one growing medium (non-soil organic substance), (iv) one soil conditioner (microbiological product formed during the compost production), (v) four preparations improving soil properties (substances added to the soil to improve its physical, chemical, and biological properties; they do not contain animal material), (vi) two manures (fertilizers of animal origin), and (vii) one agricultural digest (residue of biogas production from various raw materials or organic waste). All fertilizers used for the tests were commercially available.

- Detection of parasite eggs
  Samples were examined using the method according to Quinn et al. (1980), by own modification. The method consists of flotation of eggs in a saturated solution of NaNO_3_ preceded by mixing the sample with the Tween-20 solution. The isolated eggs of *Ascaris* spp., *Toxocara* spp., and *Trichuris* spp. were counted in each sample, then calculated per 1 kg of sample.
- Assessment of the viability of eggs of intestinal parasites of the genera Ascaris, Toxocara, and Trichuris by staining with calcein and propidium iodide

Calcein, in a living eukaryotic cell, emits green fluorescent light, and propidium iodide penetrates inside the dead cells through the damaged cell membrane and interacts with the DNA emitting red fluorescent light. Both dyes are used to distinguish between living and dead cells.

Isolated eggs on the filters were rinsed with PBS solution into a beaker. FluoCell Double Staining Kit staining solution was added, and the sample was incubated for 15 min at 37 °C in the dark; then, the sample was stored for 2 days at 6–8 °C. After filtering onto Millipore and Whatman 12-μm polycarbonate filters, the prepared slides were examined under a fluorescent microscope (470–490 nm light wavelength) (Włodarczyk et al. 2017).

## Results

**Fig. 1:**
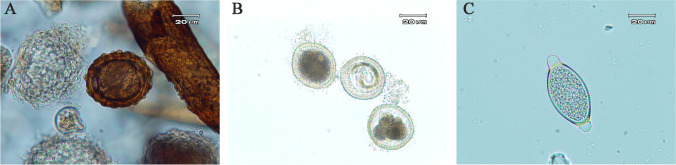
Eggs of parasitic nematodes found in commercially available organic fertilizers: **A**—*Ascaris* sp. **B**—*Toxocara* sp. **C**—*Trichuris* sp

Nematode eggs of the genera *Ascaris*, *Toxocara*, and *Trichuris* were observed in nine out of fourteen tested samples taken from the products available on the market (Fig. 1). Only in one of them the eggs were dead. Eggs containing live developed larvae were also observed. The incidence of nematode eggs in commercially available soil improvers is shown in Table 1.

**Table 1:** Occurrence of eggs of intestinal parasites in fertilizers commercially available

Sample no	Product type	*Ascaris* sp.	*Toxocara* sp.	*Trichuris* sp.
1	Organic fertilizer	-	-	-
2	Organic fertilizer	+ (1)*	-	+ (1)
3	Organic fertilizer	+ (12)	+ (20)	+ (25)
4	Organic-mineral fertilizer	-	-	-
5	Organic-mineral fertilizer	-	-	-
6	Growing medium	+ (N)*	+ (N)*	+ (N)*
7	Soil conditioner	+ (1)	+ (1)*	-
8	Preparation improving soil properties	-	-	-
9	Preparation improving soil properties	+ (N)	-	+ (N)
10	Preparation improving soil properties	-	+ (N)	+ (N)
11	Preparation improving soil properties	+ (N)	-	+ (N)
12	Manure	+ (N)	-	+ (1)
13	Manure	+ (1)*	-	+ (1)
14	Agricultural digest	-	-	-

^*^Dead eggs

() number of eggs/1 kg of product

*N*, numerous; more than 100 eggs

In the tested products, eggs of one, two, and even all three genera of nematodes were found. Live larvae of parasites were present in the soil fertilizer, in one manure, and in one of the organic fertilizers.

Most of the positive samples, as much as 50%, contained *Trichuris* sp. eggs, 36%—*Ascaris* sp., and the remaining 14%—*Toxocara* sp. (Fig. 1). The calculated number of nematode parasitic eggs per 1 kg of the product ranged from 868 to 3652.

## Discussion

Nowadays, in many countries, the basic method for management of the sewage sludge from municipal sewage treatment plants is their use for natural purposes. From the aspect of physicochemical properties, sewage sludge is similar to humus (Zdybel et al. 2019). Considering the content in sewage sludge of biogens indispensable for plants, fertilization with sludge considerably increases the crop yield (Bojarska et al. 2007; Kirchmann et al. 2017). However, the use of sewage sludge for the production of plant growth promoters brings the risk of microbiological and parasitological contamination of cultivated plants. There are known methods of devitalizing pathogens in sewage sludge before their use in agriculture or gardening (Farzadkia and Bazrafshan 2014) and include composting at high temperature, dewatering, liming, or fermentation (Capizzi-Banas et al. 2004; Manga et al. 2016; Wu et al. 2020; Liang et al. 2021). After the devitalization process, and prior to the application, sewage sludge is examined for the presence of pathogens and live eggs of intestinal parasites. However, in practice, devitalization treatments may be ineffective, particularly with respect to infective nematode eggs (Grobelak et al. 2016; Zdybel et al. 2019). Our research also showed that the diagnosis of devitalized sewage sludge is ineffective, because in products made on its basis, we found the numerous infective eggs of parasites (Table 1). *Ascaris* sp., *Toxocara* sp., and *Trichuris* sp. eggs were present in most of the products, which clearly indicates that they pose a real threat to the health of users (Else et al. 2020). In addition, there are no regulations regarding the testing of products based on sewage sludge available on the market.

In two of the three tested organic fertilizers, we found live eggs of intestinal parasites. Such contamination in fertilizers may be caused by an inadequately conducted composting process or its duration being too short. The available data show that a properly conducted devitalization process is very effective. This is indicated by the results of the studies by Wolna-Maruwka et al. (2009). The authors checked the rate of development and survival of parasites in sewage sludge during the composting process with various additives (straw, sawdust, bark, and hemp). They showed that the eggs of *Ascaris* sp., *Toxocara* sp., and *Trichuris* sp. were not present in any of the samples taken from the composter. Also, Malej (2000) confirmed that it is possible to completely eliminate pathogens and parasites from sewage sludge used for the production of organic fertilizers. The author pointed out how important it is to carry out rendering processes at high temperature for a sufficiently long time. Anaerobic stabilization at 35 °C, according to the author, is effective against the viability of intestinal parasite eggs only by 30–50%, but at 49 °C, it increases to 99%. The use of the sedimentation method removes helminth eggs up to 98%, and the use of activated sludge is effective up to 99%, liming sewage up to pH = 12—in 26.5%, and soil filters in 76%. According to Malej (2000), the complete destruction of parasites is possible after the sludge has been exposed to temperatures in the range of 62–75 °C for more than 3 weeks. Unfortunately, Gałwa-Widera et al. (2011), while examining the hygienization process of a mixture of sewage sludge and sludge from the dairy industry during composting, emphasized the catastrophically low effectiveness of treatments performed in a sewage treatment plant. Similarly, Adamus-Białek et al. (2015) found live eggs of *Ascaris* sp., *Toxocara* sp., and *Trichuris* sp. in sludge from several sewage treatment plants in Poland. Hudzik and Wodzisławska-Czapla (2011) found parasite eggs in 35 out of 546 tested sediment samples, and Siuta (2015), while examining soil samples from fields fertilized with composted sewage sludge, found parasite eggs in the amount exceeding 2000 per 1 kg of dry matter. He estimated that improper hygienization in the sewage treatment plant resulted in the introduction of nearly 3000 live helminth eggs per m^2^ into the environment.

Looking for the reasons for such varied effectiveness of hygiene treatments in sewage treatment plants, Budzińska et al. (2016) found that apart from the composting time and temperature, the survival of *Ascaris suum* eggs may be determined by their location in the compost layer. The authors unequivocally showed that the lower lying eggs require a longer time for devitalization. The introduction of detailed regulations regarding the methodology of sludge sampling for parasitological tests would allow for a reliable assessment of the sanitary condition of the tested material, and thus eliminate the risk of parasite infestation when users come into contact with products containing sewage sludge available in the market.

Our research showed that organic-mineral fertilizers were safer in terms of the presence of parasites. Nematode eggs were not found in any of the products of this type tested by us (Table 1). Hygienization based on chemical methods, such as adding quicklime to sewage sludge (Capizzi-Banas et al. 2004), granulating the sludge with potassium compounds, or adding magnesite and sulfuric acid, seems to be more effective than composting alone. These processes cause the fertilizer to be diluted by adding reactive substances. The combination of composting with chemical methods of sewage sludge sanitation significantly increases the safety of using the substrate for the production of fertilizers (Grobelak et al. 2016).

## Conclusion

Regardless of the results of scientific research on the effectiveness of methods devitalizing pathogens and parasites in sewage sludge, it is necessary to educate the society in the use of commercially available products, based on sewage sludge. Although the quality of these products should not raise any objections, their conscious use is an important supplement to health safety. It is estimated that the number of ascaridiosis cases in the world reached 804 million (Jourdan et al. 2018), the estimated global T-seroprevalence rate of toxocariasis was 19% (Rostami et al. 2019), and prevalence of trichuriasis reached 500 million (Barda et al. 2015), which indicates that intestinal helminthiasis is a significant epidemic problem. It is worth emphasizing that these numbers of infected people, especially in developed European countries, include cases of parasitic infections resulting from the use of egg-contaminated products available on the market.
